# Social Structure of a Semi-Free Ranging Group of Mandrills (*Mandrillus sphinx*): A Social Network Analysis

**DOI:** 10.1371/journal.pone.0083015

**Published:** 2013-12-10

**Authors:** Céline Bret, Cédric Sueur, Barthélémy Ngoubangoye, Delphine Verrier, Jean-Louis Deneubourg, Odile Petit

**Affiliations:** 1 Centre National de la Recherche Scientifique, Département Ecologie, Physiologie et Ethologie, Strasbourg, France; 2 Université de Strasbourg, Institut Pluridisciplinaire Hubert Curien, Strasbourg, France; 3 Unit of Social Ecology, Université Libre de Bruxelles, Brussels, Belgium; 4 Centre International de Recherches Médicales de Franceville, Franceville, Gabon; German Primate Centre, Germany

## Abstract

The difficulty involved in following mandrills in the wild means that very little is known about social structure in this species. Most studies initially considered mandrill groups to be an aggregation of one-male/multifemale units, with males occupying central positions in a structure similar to those observed in the majority of baboon species. However, a recent study hypothesized that mandrills form stable groups with only two or three permanent males, and that females occupy more central positions than males within these groups. We used social network analysis methods to examine how a semi-free ranging group of 19 mandrills is structured. We recorded all dyads of individuals that were in contact as a measure of association. The betweenness and the eigenvector centrality for each individual were calculated and correlated to kinship, age and dominance. Finally, we performed a resilience analysis by simulating the removal of individuals displaying the highest betweenness and eigenvector centrality values. We found that related dyads were more frequently associated than unrelated dyads. Moreover, our results showed that the cumulative distribution of individual betweenness and eigenvector centrality followed a power function, which is characteristic of scale-free networks. This property showed that some group members, mostly females, occupied a highly central position. Finally, the resilience analysis showed that the removal of the two most central females split the network into small subgroups and increased the network diameter. Critically, this study confirms that females appear to occupy more central positions than males in mandrill groups. Consequently, these females appear to be crucial for group cohesion and probably play a pivotal role in this species.

## Introduction

The mandrill species fascinates many, and is most probably well-known due to the striking colours males exhibit. However, surprisingly little is known about their social structure. This is due to the difficulty of locating and following them in the rainforest of Central Africa and the near impossibility of habituating mandrill groups in the wild [1], [2]. In previous studies, it was commonly accepted that mandrills presented multi-levelled social structure similar to those observed in baboon societies [3], [4], [5], [6], even if their social structures bore a closer resemblance to those seen in terrestrial mangabeys (*Cercocebus* [7]). Social structure is considered to exist when the frequency of aggression decreases and/or cohesion between group members with different interests increases [8], [9]. In the social structure of mandrills, the basal unit would be one-male/multifemale units (OMUs), also called harems [10], [11]. These OMUs occasionally form large hordes of hundreds of individuals [1], [4], [6]. In these groupings, fully-coloured adult males are considered as central individuals in the social structure and during group movements: they are dominant and are supposed to possess a greater knowledge of their environment in order to lead the group to feeding sites [4]. This type of leadership, centred on dominant and old individuals, recalls examples such as matriarchs in elephants (*Loxodonta Africana* [12]) or silverbacks in gorillas (*Gorilla gorilla* [13]). Moreover, previous studies have highlighted that dominant males are often larger and heavier than the rest of the group (males are 3.4 times heavier than females [14]), and thus have greater nutritional needs. These energetic demands are reported to influence the number of initiations of group movements [15], [16] and it is logically assumed that adult males are central and lead groups.

More recently, a completely different description of mandrill organization was proposed. Abernethy et al [2] suggested that large groups of mandrills are not occasional OMU aggregates, but are rather permanent groups. Indeed, like in sooty mangabeys (*Cercocebus atys* [17]), only a low number of adult males are stable members of these groupings, together with adult females and their offspring, while other adult males only enter the groups during the breeding season [2]. Thus, the presence of these permanent and affiliated males seems to be a sign of stability in mandrill groups, where individuals have long-term and differentiated social relationships [2], [18]. Additionally, Abernethy et al [2] suggested that females would occupy a more central position in the group than males [10], given the highly biased sex-ratio in this species. However, these studies were based solely on the demographic analysis of group composition, carried out during opportunistic observations of wild mandrill groups. Hence, a meticulous social structure analysis of identified groups is necessary to investigate organization in mandrills.

We therefore used social network analysis methods to understand the organization of a small semi-free ranging group of mandrills. These methods are recognized as a valuable tool to study social complexity [19], [20], [21]. Social network analysis has already provided new insights into the social structure of numerous mammal species. These studies relied on different kinds of observations like social interactions (pigtailed macaques *Macaca nemestrina* [22]; killer whales *Orcinus orca* [23]; Columbian ground squirrels *Spermophilus columbianus* [24]; yellow-bellied marmots *Marmota flaviventris* [25]; chimpanzees *Pan troglodytes* [26]), or associations between individuals (i.e. individuals observed together or within a specified distance; bottlenose dolphins *Tursiops truncates* [27]; spider monkeys *Ateles geoffroyi* [28]). Moreover, previous studies have quantified differences in social structure in phylogenetically close species (onagers *Equus hemionus* & Grevy's zebras *Equus grevyi* [29]; rhesus macaques *Macaca mulatta* & Tonkean macaques *Macaca tonkeana* [9], [30]), showing that social network analysis methods give efficient and accurate results. Social network analysis was also used to explore the stability of the social structure of chacma baboons over time (*Papio ursinus* [31]). Researchers recently investigated the possibility of key individuals existing in social groups, and the role these individuals may play in group cohesion [32], [33]. To do so, they studied the impact on cohesiveness when these key individuals disappeared by removing them from the group either experimentally [23], [34] or theoretically [35]. In this context, a group is considered to have remained cohesive if the removal of one or more randomly chosen individuals does not split the group into subgroups [36], [37].

In the present study, we investigated the social structure of a semi-free ranging colony of 19 mandrills, one of three mandrill colonies located at the International Center for Medical Research in Franceville (CIRMF), Gabon. Indeed, most of our knowledge of mandrill behaviour was gained through observation of these colonies. Their demographic properties are similar to those of wild groups, with most adult males occupying peripheral positions, mimicking migration, as defined by Setchell & Dixson [2], [38]. The social structure was analyzed using social network analysis methods through an association measure based on *body contacts*. We also tested whether kinship, age, dominance or sex impact the distribution of relationships among group members. Finally, we investigated whether there were key individuals among group members, evaluated their rank and identity, and determined what specific role, if any, they played in group cohesion. We hypothesized that females would have a more central position than males, according to the biased sex-ratio described in mandrills. We expected these central females to have an important role in group cohesion.

## Methods

### Ethics statement

Our methodological approach solely involved observations. Animals were not handled, and no invasive experiments were carried out. Our protocol followed the ethical guidelines of our institution and the recommendations of the Gabonese government. This study was conducted with the approval of the CIRMF scientific committee in Gabon via a research agreement (n°045/2011/CNRS). All occurrences of injuries or illness in the observed animals were reported to veterinary staff at the CIRMF primatological center. Animals were already used to human presence in their enclosure.

### Study group and environment

The study was carried out from April to August 2011 on a group of 19 mandrills born in captivity and living in a naturally rainforested enclosure (1.5 ha), at the CIRMF, Gabon. This colony was established between 2002 and 2008 by transferring 16 individuals from the two other enclosures (see Table 1 for transfer dates). The history of the previous colonies is described by Setchell et al [39]. Any increases in group size through natural reproduction were counterbalanced by deaths or the permanent removals of individuals for management purposes. The group foraged freely in the enclosure, and was supplied with home-made soya-cake and local seasonal fruits twice a day. Water was available *ad libitum*. The group was composed of 4 fully-developed adult males, 13 adult females, 1 subadult male and 1 juvenile female (see Table 1 for details about individuals). Age classes were based on previous studies on captive mandrills [38], [40]. For this study, only adult and subadult individuals were included for the analyses, as juveniles spent all their time with their mothers, and their relationships with other group members were not stable [37]. The CIRMF colonies have been followed since the foundation of the first group in 1984. Dates of birth and motherhood were recorded for all the individuals. All subjects were identified using morphological differences and/or ear tags. The group was observed 4–5 h per day (from 06:00 am to 11:00 am) by one observer (C.B.) within the enclosure, and for 1 h after food delivery (from 11:30 am to 12:30 am) by the same observer located outside the enclosure. Within the enclosure, C.B. remained with the group and only changed her position when all visible individuals went elsewhere. C.B. was trained to identify the different group members.

**Table 1:** Individual details about sex (M =  male, F =  female), class (A =  adult, SA =  sub-adult, J =  juvenile), age, matriline, dominance rank and transfer date of the study group.

ID	Sex	Class	Age	Matriline	Rank	Transfer date
5i1	M	A	10.5	5	1	Aug 2002
5F	M	A	21	5	2	June 2007
5J1	M	A	10	5	3	Aug 2002
12A3D	M	A	10	12	6	Aug 2002
12M2	M	SA	8.5	12	14	Fev 2003
12D4	F	A	17	12	4	Aug 2002
12D4A	F	A	8	12	5	*
12A7	F	A	17	12	18	May 2009
12A7A	F	A	8.5	12	9	Aug 2002
12A5	F	A	19	12	15	Sept 2002
2D4E	F	A	6	12	13	Apr 2005
2D4E1	F	J	1	12	16	*
10A	F	A	27	10	11	Sept 2002
10A6	F	A	9.5	10	12	Sept 2002
10A1	F	A	22	10	10	Aug 2002
10A1D	F	A	10	10	8	Aug 2002
2i	F	A	19	2	7	Sept 2002
28A	F	A	8	28	17	*
5A5	F	A	15.5	5	19	Oct 2008

Individuals with identical numbers in the matriline column belong to the same matriline.

Individuals born in the enclosure.

### Data collection

The instantaneous scan sampling method [41] was used to record the position of each visible individual inside the enclosure every 15 minutes. We collected 479 scans from the enclosure during the observation period. We then constructed a matrix considering the number of scans each time a dyad was seen to be in contact (*contact* matrix).We calculated a half-weight association index (HWI) for each pair of individuals: 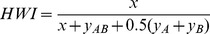
where 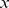 is the number of scans where A and B were observed associated, 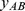 the number of scans where A and B were not associated, 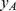 the number of scans with A only, and 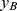 the number of scans with B only [42]. As we did not observe the entire group at each scan, the individuals were not all observed at the same frequency (chi-square test: χ*^2^* = 456.98, *df* = 17, *P*<0.01). The half-weight association index therefore allowed us to control for absences. We then visualized the *contact network* with Gephi 0.8.1 (Gephi Consortium 2008, [43]).

**Figure 1:**
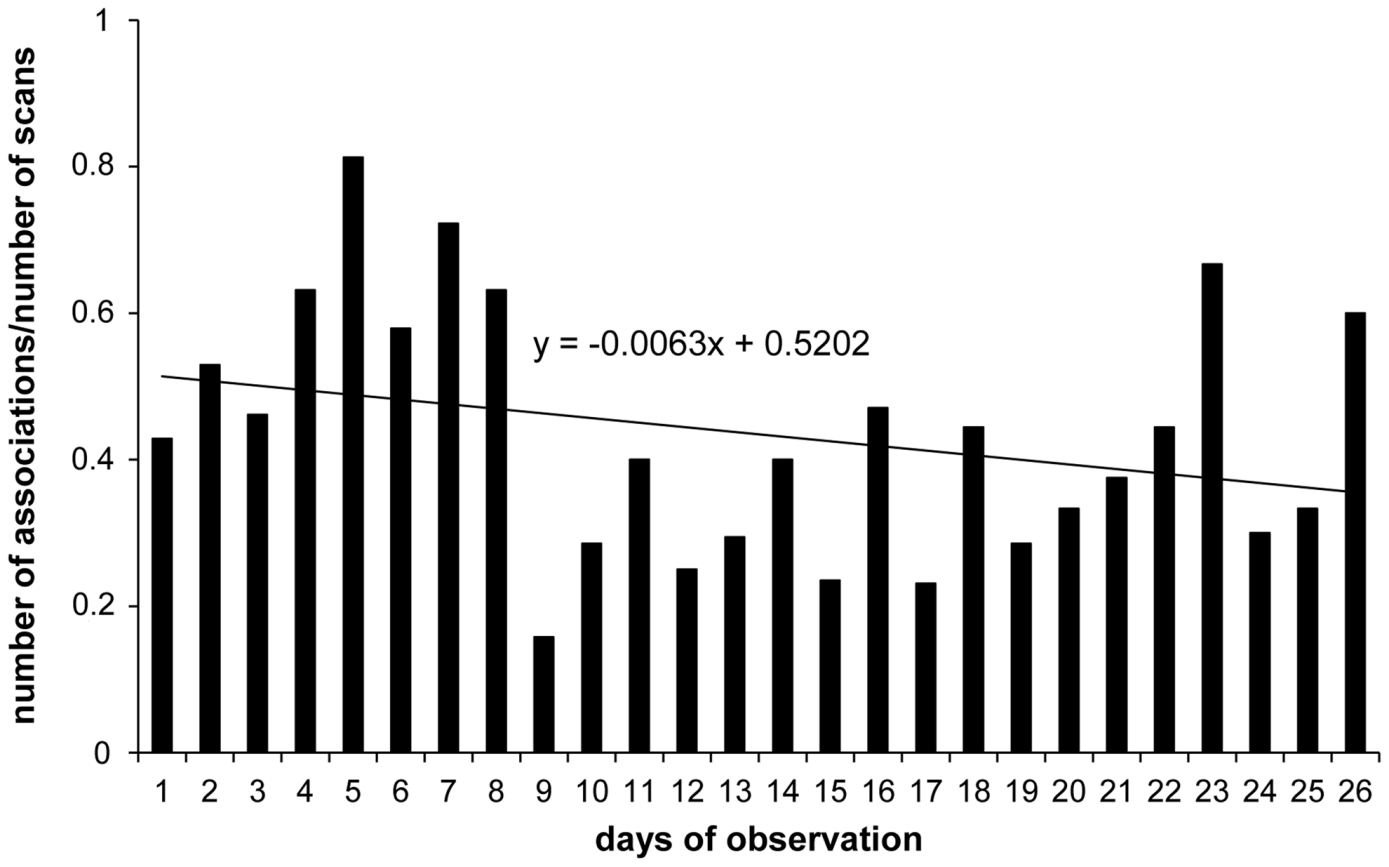
Number of associations observed per day corrected by the number of scans recorded per day. The solid line represents the trend followed by the distribution over time. The slope of this linear curve was not statistically different from 0, meaning that the distribution of the number of associations corrected by the number of scans did not significantly evolve over time.

In order to investigate the stability of the network over time, the whole dataset was split into two equal periods: from April to mid-June and from mid-June to August. The comparison of association matrices for the two periods using the Dietz'R matrix correlation test implemented in Socprog 2.4 [44] revealed the two matrices to be significantly correlated (Dietz'R matrix correlation: *R* = 0.249, *P*<0.01), meaning that the social relationships observed during the two periods were similar. We also found that the number of associations observed per day corrected by the number of scans recorded per day was homogeneous over time (Fig. 1, slope statistically not different from 0, linear regression, *t* = −1.462, *P*>0.05). In conclusion, the network was stable over the entire observation period (April to August) and this allowed us to consider the whole dataset for the subsequent analyses.

We established the dominance/subordination hierarchy of the group by recording spontaneous agonistic events (all-occurrences sampling, [41]). Socprog 2.4 was used to determine individual rank. This hierarchy was significantly linear (1000 permutations, *P*<0.001, *h′* = 0.632, [45]), and also significantly unidirectional (Dietz'R matrix correlation, *r* = −0.531, *P*>0.05). We then constructed a hierarchy matrix, representing the rank difference within each dyad.

Finally, we constructed matrices of sex, age and kinship. In the sex matrix, dyads of the same sex were coded 1 and dyads of different sex were coded 0. The age matrix was constructed using the age difference for each dyad. In the kinship matrix, all the related dyads were coded 1 and the unrelated dyads were coded 0. We considered a dyad as related when the two individuals belong to the same matriline. We tested the correlations between kinship, sex, age and hierarchy matrices using the Dietz'R matrix correlation test: none was statistically significant (Dietz'R matrix correlations: *R*<0.23, *P*>0.05).

### General networks properties

Our first step was to analyze the global properties of the *contact network*. We achieved this by calculating the mean degree and the mean global clustering coefficient (see Table 2 for definitions). The network diameter and the density (see Table 2 for definitions) were also calculated.

**Table 2:** Network analysis measure definitions.

Measures	Definition	Reference
Mean degree	The mean number of individuals connected to an individual	Wasserman & Faust [46]
Mean clustering coefficient	The mean degree to which the associates of an individual are associated amongst themselves	Whitehead [42]
Diameter	The longest shortest path from any given individual to another in the network	Wasserman & Faust [46]
Density	The fraction of observed relationships among all possible relationships	Wasserman & Faust [46]
Betweenness	The number of shortest paths between pairs of individuals that pass through the individual in question	Whitehead [42]
Eigenvector centrality	The number and strength of relationships between group members and the considered individuals	Whitehead [42]
Fragmentation	The number of individuals disconnected from the main subgroup divided by the group size	Wasserman & Faust [46]

In order to define which parameters could explain the observed relationships distribution, we investigated the correlation between the *contact* association matrix and those of kinship, hierarchy, sex and age using the Dietz'R matrix correlation test. For each correlation, we performed 10,000 permutations to obtain more stable and accurate p-values [47]: lines and columns were permuted randomly 10,000 times in order to obtain random matrices. The statistic obtained from the real matrix was then compared to statistics obtained from the random matrices, and if the real statistic was less or greater than the random value for 97,5% of the random permutation, the test is considered significant. We then identified clusters of individuals showing the strongest relationships in the *contact network* through hierarchical cluster analysis with the modularity 1 option (based on the difference between the proportion of the total association within clusters and the expected proportion, calculated by the sum of the associations of the different individuals) in Socprog 2.4 [48]. The average linkage option was used in order to provide a better cophenetic correlation coefficient (cophenetic correlation coefficient >0.8). We then built a matrix where dyads belonging to the same cluster were coded 1 and dyads belonging to different clusters were coded 0. We further investigated the correlation between clusters, hierarchy, kinship and age matrices using the Dietz'R matrix correlation test.

### Individual roles and network cohesion

In the second part of the study, we investigated the role played by the different group members in the *contact network*. We first calculated the betweenness and the eigenvector centrality for each individual using Gephi 0.8.1 and Socprog 2.4 (see Table 2 for definitions and Table 3 for individual details). Betweenness and eigenvector centralities are the most appropriate centrality measures for our study, as they reflect the connectivity and social centrality of individuals in networks [49]. Moreover, we found no correlation between these two measures (Spearman rank correlation test: *r* = 0.423, *P* = 0.08, *N* = 18), so we retained both indexes for further analyses. Curve estimation tests were used to compare the cumulative distribution of betweenness and eigenvector centralities to a linear function, characteristic of a random network, also called the Erdös-Rényi network [50], and to a power function, characteristic of a scale-free network [51]. For each test, the Akaike Information Criterion (AIC) was calculated using the residual sum of squares (RSS):
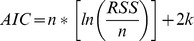
where 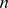 is the number of observations and 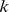 is the number of degree of freedom. Although individuals have different centralities in both random and scale-free networks, these differences are stronger in scale-free networks with some highly central individuals. These central individuals were isolated using the *influence.measures* test developed in R by Fox [52].

**Table 3:** Individual details of degree, eigenvector centrality and betweenness for the *contact network*.

ID	Degree	Eigenvector centrality	Betweenness
5i1	3	0.16	11.283
5F	3	0.38	1.583
5J1	2	0.08	0.5
12A3D	0	0	0
12M2	3	0.11	3.25
12D4	7	0.62	26.533
12D4A	4	0.47	6.583
12A7	2	0.2	0
12A7A	3	0.03	9.017
12A5	0	0	0
2D4E	5	0.19	13.867
10A	3	0.23	1.917
10A6	5	0.25	10.017
10A1	4	0.03	26.5
10A1D	2	0.01	13
2i	3	0.08	8.95
28A	1	0	0
5A5	0	0	0

The betweenness and the eigenvector centrality were correlated with dominance, age and number of kin. Spearman rank correlation tests were used for all these comparisons.

We then investigated the role of central individuals on the stability of the *contact network*. A network was defined as stable if the removal of an individual did not affect the network structure. To do so, we simulated 1) the removal of individuals with the highest betweenness and eigenvector centrality values (targeted condition), and 2) the removal of randomly chosen individuals (random condition), using the techniques described by Lusseau [35]. Random removals were repeated ten times [24], [26]. This method allowed us to evaluate the importance of central individuals on group cohesion. This was tested through the investigatation of changes in the network fragmentation and diameter (see Table 2 for definitions). We analyzed these changes between targeted and random conditions.

All statistical tests were carried out with SPSS 17.0 and R 2.8.1, with α = 0.05.

## Results

### Network structure

**Figure 2:**
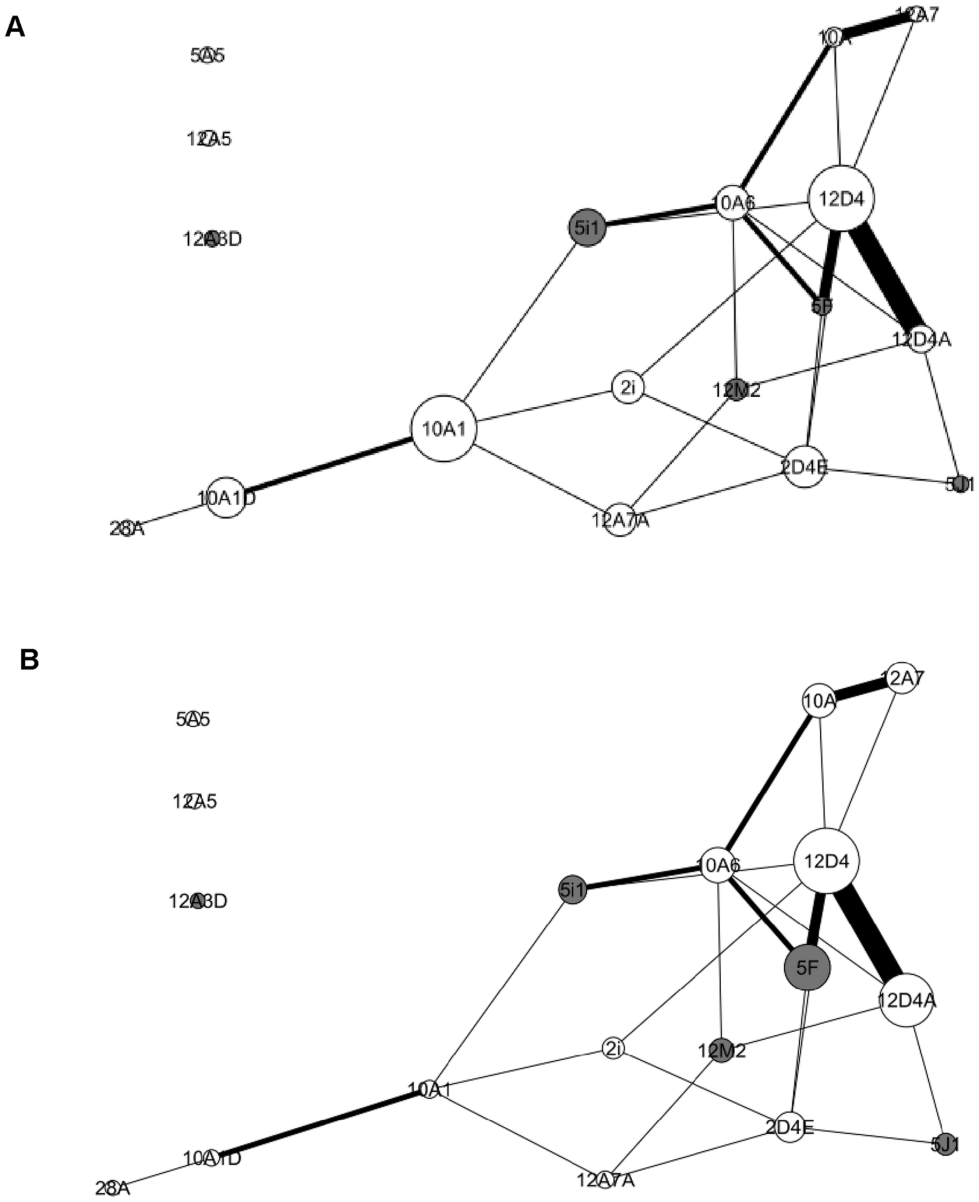
Representation of the *contact network*. Nodes represent individuals, and the size of nodes is related to the individual's betweenness (A) and the individual's eigenvector centrality (B), with bigger nodes corresponding to more central individuals. White nodes correspond to females and dark gray nodes correspond to males. Widths of lines represent the strength of association between two individuals.

We first investigated the general properties of the *contact network* presented in Figure 2, constituted of twenty-five of the one hundred and fifty-three possible edges (density  = 16.3%). The *contact network* had a diameter of 3 and a mean degree of 2.78±1.86. Thus, this network had a low density and is little connected. Moreover, the average clustering coefficient was 0.039±0.059, meaning that individuals connected to a specific group member are not connected among themselves.

We then analyzed the relationship distribution in the *contact network*. When solely females were considered, kin-related dyads were more frequently associated than unrelated dyads (Dietz'R matrix correlation: kinship: *r* = 0.184, *P* = 0.05; entire group: *r* = 0.072, *P* = 0.182). The association matrix did not correlate with age, sex and dominance (Dietz'R matrix correlation: age: *r* = 0.057, *P* = 0.294; sex: *r* = 0.001, *P* = 0.99; dominance: *r* = −0.088, *P* = 0.767). We also identified nine clusters in the *contact network* (maximum modularity  = 0.379, cophenetic correlation coefficient  = 0.843). Nonetheless, clusters were not defined by age, hierarchy or kinship (Dietz'R matrix correlations, age: *r* = 0.045, *P* = 0.326; hierarchy: *r* = −0.11, *P* = 0.895; kinship: *r* = 0.042, *P* = 0.507).

### Individual roles

**Figure 3:**
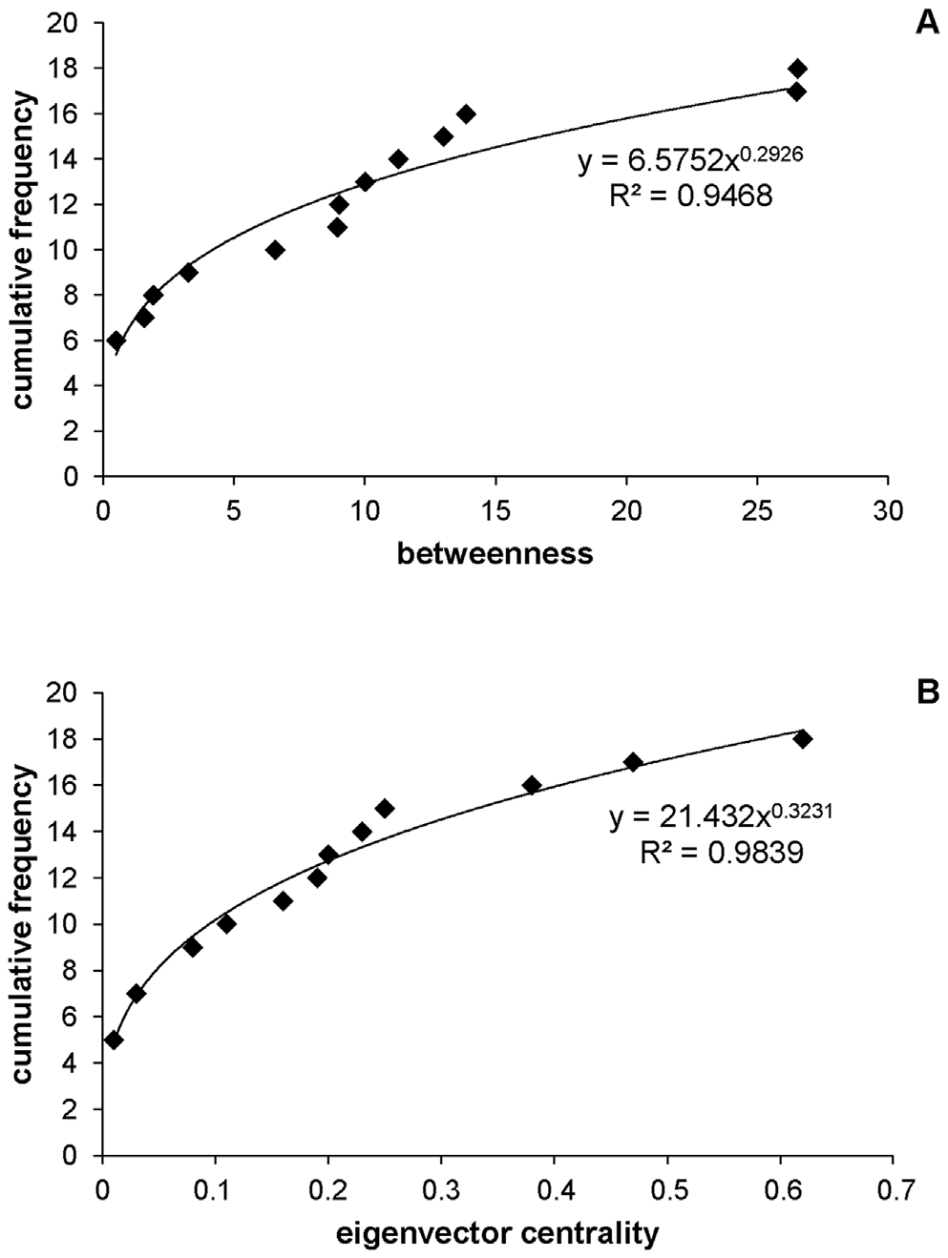
The cumulative distribution of centrality values for the *contact network*. (A) Betweenness values, and (B) eigenvector centrality values. Solid lines represent the power function fitted by the distributions.

In order to highlight if some individuals displayed high centrality values, we determined if our network was random or scale-free. To do so, we analyzed the distribution of betweenness and eigenvector centrality. The cumulative distribution of betweenness values fitted a power function (power curve estimation test: *AIC* = −0.428, 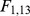 = 402.34, *P*<0.01, Fig. 3A; linear curve estimation test: *AIC* = 9.741, 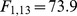, *P*<0.01), as well as the cumulative distribution of eigenvector centrality values (power curve estimation test: *AIC* = −11.091, 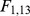 = 612.11, *P*<0.01, Fig. 3B; linear curve estimation test: *AIC* = 10.883, 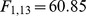, *P*<0.01). Thus, the *contact network* was more similar to a scale-free network than to a random network for both centrality measures, meaning that some individuals had a higher centrality than the other members of the group. Indeed, figure 3A and *influence.measures* test highlighted that only two individuals presented a high betweenness value. These two individuals were both females (Fig. 3A, individuals 10A1 and 12D4). When looking for a specific status for these individuals, we found that 12D4 was the dominant female, and 10A1 was a member of the largest direct matriline (Table 1). In figure 3B and in the *influence.measures* test, three individuals presented a higher eigenvector centrality value than the rest of the group: two females, 12D4 and 12D4A, and one male, 5F. The two females were the dominant females and 5F was the oldest male in the group (Table 1).

In addition, we found a significant correlation between betweenness and dominance when solely females were considered, meaning that central females were also high-ranking females (Spearman rank correlation test: *r* = −0.657, *P*<0.05, *N* = 13). We also found a tendency for central females (in term of eigenvector centrality) to be high-ranking females, but the correlation was not significant (Spearman rank correlation test: *r* = −0.534, *P* = 0.06, *N* = 13). These results confirmed our previous findings for the identity of two central females (12D4 and 12D4A). Finally, neither age nor number of kin were correlated to both centrality measures (Spearman rank correlation test - betweenness: age: *r* = −0.054, *P* = 0.859, *N* = 13; kinship: *r* = 0.129, *P* = 0.675, *N* = 13; eigenvector: age: *r* = −0.008, *P* = 0.978, *N* = 13; kinship: *r* = 0.353, *P* = 0.236, *N* = 13).

### Network stability

**Figure 4:**
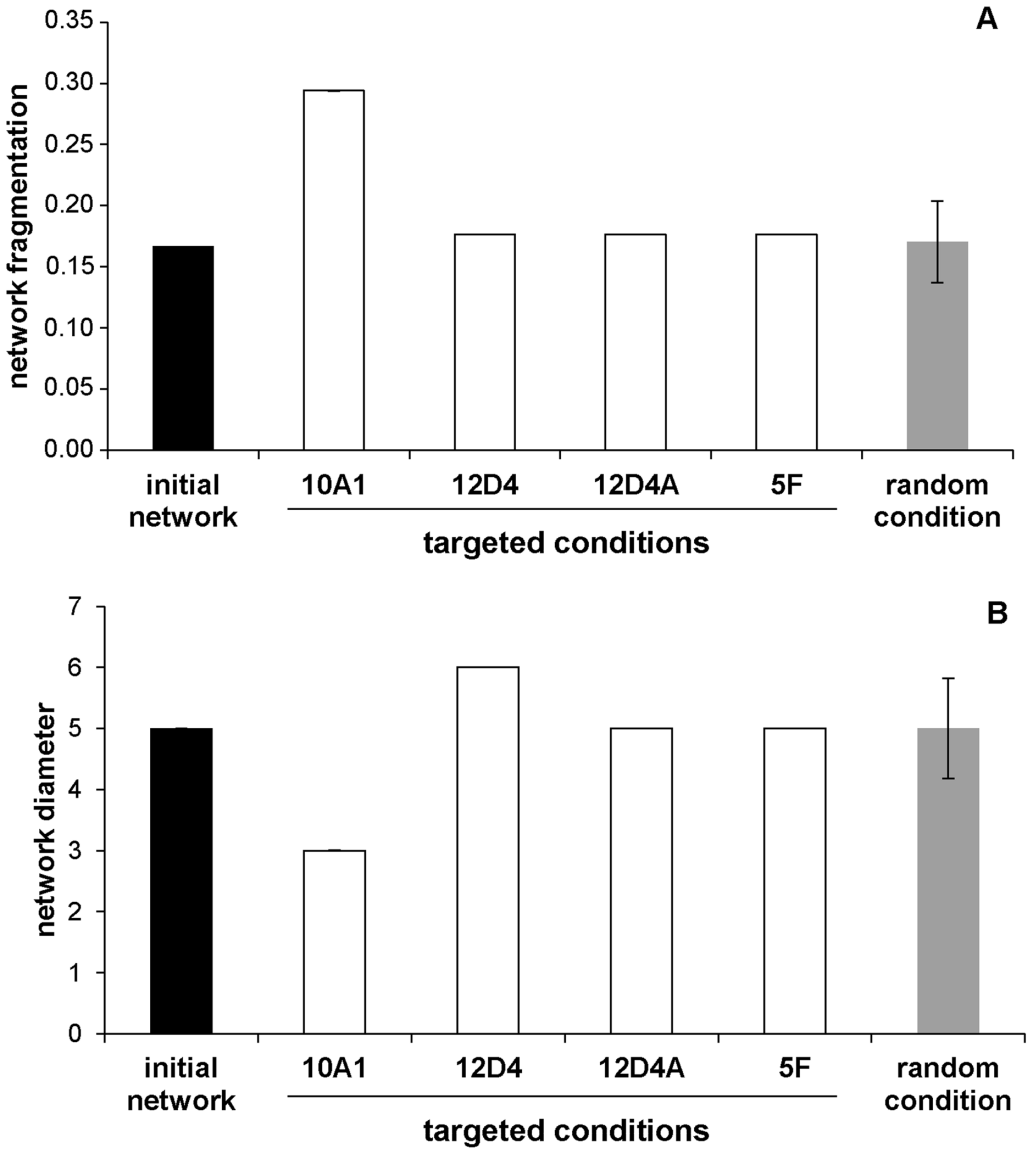
Changes in network characteristics after the removal of central individuals (targeted condition) and randomly chosen individuals (random condition). (A) Network fragmentation, and (B) network diameter. In all figures, dark columns represent the initial network, grey columns represent random condition and white columns represent targeted condition.

In this analysis, we evaluated the weight of the most central individuals (those with high betweenness and eigenvector centrality) in network stability by removing them from the *contact network*. We first focused on changes in the network fragmentation (Fig. 4A). After the removal of 10A1, one of the females displaying a high betweenness value, the fragmentation *f* of the *contact network* increased (initial network: *f* = 0.167, removal of 10A1: *f* = 0.294). In other words, the number of individuals disconnected from the main subgroup increased. The other targeted removals (of 12D4, 12D4A and 5F) and random removals did not affect network fragmentation (initial network: *f* = 0.167, targeted and random removals: *f* = 0.176, same result for all the analyses).

We then investigated changes in the network diameter (Fig. 4B). Consistently with previous results, after the removal of 10A1, the network diameter *d* decreases (initial network: *d* = 5, removal of 10A1: *d* = 3). Indeed, as the number of individuals which remained connected in the main subgroup decreased, so did the distance between individuals. We also found that the diameter increased after the removal of 12D4, another central female in terms of betweenness and eigenvector centrality (initial network: *d* = 5, removal of 12D4: *d* = 6). Members of the *contact network* were then less connected. The other targeted removals (of 12D4A and 5F) and random removals did not affect network diameter (initial network: *d* = 5, targeted and random removals: *d* = 5, same result for all the analyses).

## Discussion

In primate species, the role played by the different group members will be different according to the type of social structure concerned [10], [53]. In this study, we explored the social structure of a mandrill group and highlighted the role of the different group members in maintaining network stability and group cohesion.

We first found a correlation between associations in dyads and kinship in females: related females associated more frequently, as previously described by Setchell [54]. This link between association and kinship was previously observed in other primate societies presenting a multi-male/multi-female structure (Japanese macaques *Macaca fuscata* and rhesus macaques [55], [56], chacma baboons [57]). In the wild, males disperse and only female mandrills remain in their birth group [2], [4]. Establishing strong relationships with their relatives could provide females with benefits such as support during conflicts [58], better survival of offspring [59] or a longer lifespan [60].

Conversely, neither kinship, hierarchy nor age were correlated to *contact* clusters. Our results suggested that females were more closely associated with kin in this studied group, while the structure of clusters was not explained by the other factors we tested. Nonetheless, this conclusion is not totally consistent with previous studies showing that females were mainly grouped in matrilines in semi-free ranging mandrills [54]. The composition of our study group could however explain this result. Indeed, some females were the only representatives of their matriline. In the same subgroups, there were therefore related females and kin-isolated females, and this could have diminished the kinship influence on subgroup composition within the study group. We therefore need to observe bigger groups with larger matrilines in order to confirm our conclusions about the factors influencing social structure in mandrills.

We investigated the possibility of central individuals in our network using curve estimation tests. These tests revealed that the betweenness distribution and the eigenvector centrality distribution were more similar to a scale-free [61] than a random network [50]. This result demonstrates that some individuals in the networks were better and more strongly connected than the rest of the group. Previous studies of the social networks of other primate groups (see Kasper & Voelkl for a review [62]) did not show primate networks to possess scale-free properties, despite the fact that these authors focused on central individuals when searching for specific roles played in the group. However, our results are supported by the description of scale-free properties in some small social mammal networks such as bottlenose dolphins [35], killer whales [23] or Columbian ground squirrels [24]. Although James et al [63] suggested that scale-free networks could not be found in small social groups, Kanngiesser et al [26] have highlighted that both scale-free and random networks could be theoretically simulated for small sample size (N = 15). This study reveals the first evidence for the existence of scale-free networks in primate social groups and suggests that central individuals are highly connected in this species (*i.e.* central individuals are more connected than in a random network).

When considering betweenness values, we found two females to be central: the dominant female 12D4, and 10A1, a female member of the largest direct matriline in the group. In addition, for eigenvector centrality values, we found three central individuals: the two dominant females and the oldest male of the group. Highly-connected individuals were mostly females, which confirm Abernethy et al. 's hypothesis [2] on sex differences in mandrill's social structure: some females are more central and might have a more important social role in the group than males. This might also be due to the dispersal of males [2], [4], which prevents them from developing relationships as strong and stable as those developed by females [10], [11].

In order to evaluate the importance of these central individuals for the stability of the network, we simulated the removal of individuals with the highest betweenness and/or eigenvector centrality values (targeted attack) and random individuals (random attack), following the method previously described by Lusseau [35]. These removals simulated the disappearance or death of individuals. We found that out of four central individuals, only two females seemed to have an impact on network stability. Indeed, after the removal of 10A1, the *contact* network was more fragmented than after other removals. As the diameter decreased, so did the number of individuals connected in the main subgroup. Our results showed that this female seem to be responsible for group cohesion. When looking more closely at the network, we found that 10A1 was the only group member that was linked to peripheral individuals. Thus, this female appeared to cement the bonds between these peripheral individuals and the main subgroup. After the removal of the dominant female 12D4, the network diameter increased, meaning that individuals were less connected. However, contrary to results after removing the old female, group fragmentation did not increase. This female therefore seemed to play an important role in the connection between individuals in the main subgroup. In conclusion, these two highly-central females seemed to have a key role for the network stability and group cohesion as a whole. It is important to note that our results were consistent with previous studies showing that interaction networks were sensitive to the removal of central individuals in other social species (pigtail macaques [22], Columbian ground squirrels [24], and chimpanzees [26]).

We are aware that our study focused on a single small mandrill group bred in semi-free ranging conditions. However, given the fact that mandrills are difficult to follow in the wild, quantitative studies are mainly carried out in captive colonies. Furthermore, the demographic properties of our study group were similar to those of wild groups, with most adult males occupying peripheral positions, mimicking migration [2], [38]. This reinforces our conviction that our findings make a valuable contribution to our understanding of mandrill social structure.

In conclusion, this study shows that females occupy a central position in mandrill social structure: they seem to play a pivotal role and appear to be crucial for group cohesion. As in other species, we might assume that these females probably contribute to the management of group conflicts [22], [64] and the efficient transfer of information between group members [65]. These results support Abernethy et al. 's [2] hypothesis concerning the identity of central individuals: females occupy more central positions than males. Thus, whilst classical behavioral analysis suggested that males occupying central positions in OMU groups [4], social network analysis seems to reveal a group organization in the multi-male/multi-female groups that is centered on females. However, this needs to be confirmed by further studies in bigger groups, and more detailed studies should be carried out in wild groups before making any conclusions about the social structure that characterizes mandrills. It would be also interesting to study the identity of central individuals in other female philopatric species that display OMU structure, such as geladas [66], [67]. Moreover, as central individuals seem to occupy a key position for group cohesion, they could play a crucial role in information transfers in processes such as collective decision-making events, as suggested by Sueur et al [33]. Understanding the role of these pivotal females in decision-making in mandrills will therefore be the next step in our work.
